# Interfacial modulation and optimization of the electrical properties of ZrGdO_*x*_ composite films prepared using a UVO-assisted sol–gel method

**DOI:** 10.1039/d4ra07704k

**Published:** 2025-01-23

**Authors:** Chaozhong Guo, Kamale Tuokedaerhan, Xiangqian Shen, Yerulan Sagidolda, Zhambyl Azamat

**Affiliations:** a Xinjiang Key Laboratory of Solid State Physics and Devices, Xinjiang University Urumqi Xinjiang 830046 China kamale9025@163.com; b The School of Physics Science and Technology, Xinjiang University Urumqi Xinjiang 830046 China; c Department of Solid State Physics and Nonlinear Physics, Faculty of Physics and Technology, AL-Farabi Kazakh National University Almaty 050040 Kazakhstan

## Abstract

In this paper, Gd-doped ZrO_2_ gate dielectric films and metal-oxide-semiconductor (MOS) capacitors structured as Al/ZrGdO_*x*_/Si were prepared using an ultraviolet ozone (UVO)-assisted sol–gel method. The effects of heat treatment temperature on the microstructure, chemical bonding state, optical properties, surface morphology and electrical characteristics of the ZrGdO_*x*_ composite films and MOS capacitors were systematically investigated. The crystalline phase of the ZrGdO_*x*_ films appeared only at 600 °C, indicating that Gd doping effectively inhibits the crystallization of ZrO_2_ films. Meanwhile, as the heat treatment temperature increased from 300 °C to 600 °C, the content of oxygen vacancies decreased from 18.57% to 11.95%, and the content of metal–hydroxyl–oxygen bonds decreased from 14.72% to 8.64%. Heat treatment temperature proved to be effective in passivating the oxygen defects and reducing the trap density within the dielectric layer. At 500 °C, the MOS capacitor exhibited the best electrical characteristics, including the highest dielectric constant (*k* = 19.3), the smallest hysteresis (Δ*V*_fb_ = 0.01 V), the lowest boundary trapping oxide charge density (*N*_bt_ = 2.7 × 10^10^ cm^−2^), and the lowest leakage current density (*J* = 9.61 × 10^−6^ A cm^−2^). Therefore, adjusting the heat treatment temperature can significantly improve the performance of ZrGdO_*x*_ composite films and capacitors, which is favorable for the application of CMOS devices in large-scale and high-performance electronic systems.

## Abstract 2

Structural diagram of ZrGdO_*x*_ gate-dielectric films based on ultraviolet ozone (UVO)-assisted treatment, and dielectric characteristics exhibited by MOS capacitors based on ZrGdO_*x*_ gate-dielectric films.

## Graphical abstract

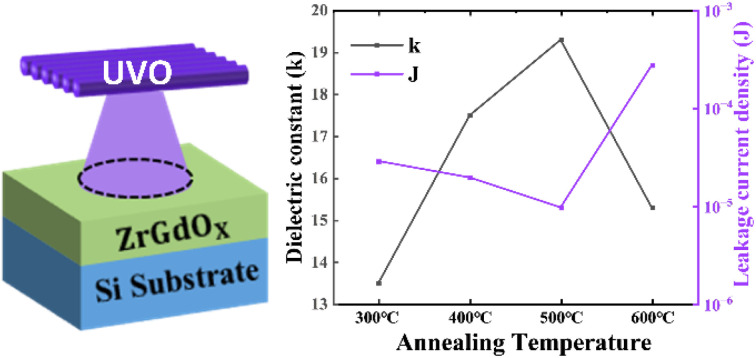

## 1. Introduction

According to Moore's law, chip integration doubles every 2–3 years, and the feature size of devices shrinks to 70% of its original size every 3 years.^1,2^ The traditional gate dielectric material SiO_2_ has been widely used due to its abundant raw materials and good thermodynamic stability in contact with Si. However, as device feature sizes continue to shrink, the physical thickness of SiO_2_ is approaching its limit, leading to increased gate leakage current and power consumption. Intel Corporation proposed using high-*k* gate dielectric materials combined with metal gate electrodes instead of SiO_2_ to address these issues.^3^ Among the various high-*k* materials, ZrO_2_ is gaining popularity due to its high dielectric constant (*k* ∼25), sufficiently large forbidden bandwidth (4.7–7.8 eV), and energy band offset (>2 eV), as well as its good thermo-chemical stability with the Si substrate during thin-film deposition and high-temperature treatment.^4–6^

However, ZrO_2_-based metal-oxide-semiconductor (MOS) capacitors face several challenges, including a low crystallization temperature, a high content of oxygen vacancies, hydroxyl oxygen and other defects. These unfavorable factors increase the trap charge density, gate leakage current, and power consumption, reducing the stability of MOS devices. To enhance the performance of ZrO_2_ capacitors, researchers have proposed various methods, such as doping with Al,^7^ Ti,^8^ Ni,^9^ or using special processes like infrared radiation^10^ to modify the ZrO_2_ gate dielectric films. As shown in Table 3, the dielectric properties of high-*k* materials modified by various process methods in recent years are summarized. Among those dopants, Gd_2_O_3_ is widely used in dielectric materials, metal oxide semiconductors, and capacitors, demonstrating unique properties that make it stand out.^11^ First, Gd_2_O_3_ has a relatively high dielectric constant (*k* = 12–16), which can enhance the dielectric constant of ZrO_2_ materials, increasing capacitance and reducing leakage current. This is particularly advantageous for miniaturizing electronic devices while maintaining or improving their performance. Additionally, Gd_2_O_3_ exhibits excellent thermal stability, which is crucial during high-temperature processing and for long-term device stability. Furthermore, Gd_2_O_3_ has a high bandgap (∼5.0 eV), which suppresses carrier transport, reduces tunneling current, and increases the breakdown voltage of the dielectric layer. Finally, doping with Gd_2_O_3_, a lanthanide rare earth oxide, can reduce defect density (*e.g.*, oxygen vacancies) and improve the interfacial quality of the dielectric layer.^12,13^ These properties make Gd_2_O_3_ an ideal dopant for dielectric materials. Therefore, this paper investigates the use of Gd_2_O_3_ as a dopant in ZrO_2_ thin films.

Currently, there are various thin film deposition techniques, including atomic layer deposition (ALD),^14^ molecular beam epitaxy (MBE),^15^ magnetron sputtering,^16^ and sol–gel method.^17^ Compared to techniques requiring expensive equipment, high vacuum conditions, and slow deposition rates, the sol–gel method offers advantages such as simplicity, high throughput, low cost, and the ability to be prepared in an air environment. This method also allows for easy adjustment of the precursor composition.^18^ However, films prepared by the sol–gel method often have poor purity and more defects. Fortunately, ultraviolet ozone (UVO) treatment can improve the quality of these films by decomposing organic matter, cleaning the surface, and obtaining a smooth film. UVO treatment also promotes the decomposition of precursors to form the metal–oxygen–metal (M–O–M) lattice and removes defects (oxygen vacancies and organic groups) through the oxygen radicals in the UVO atmosphere, thereby densifying the films.^19–21^

In this paper, a simple and low-cost UVO-assisted sol–gel method is proposed to prepare high-performance ZrGdO_*x*_ composite gate dielectric films. The effects of different annealing temperatures on the microstructure, chemical bonding state, optical properties, and surface morphology of ZrGdO_*x*_ composite films are systematically investigated. Additionally, MOS capacitors with the structure of Al/ZrGdO_*x*_/Si were prepared to study the electrical characteristics of the devices at different annealing temperatures and to analyze the gate leakage current conduction mechanism. The results show that the ZrGdO_*x*_ composite films annealed at 500 °C exhibit the best dielectric properties, supported by various characterization methods. A comparison with some high-*k* materials reported in recent years is also presented, as shown in Table 3.

## 2. Experimental

### 2.1 Preparation of ZrGdO_*x*_ precursor solution

ZrGdO_*x*_ precursor solutions were prepared by dissolving zirconium chloride octahydrate (ZrOCl_2_·8H_2_O, Aladdin, 99%) and gadolinium nitrate hexahydrate (GdN_3_O_9_·6H_2_O, Aladdin, 99.9%) in 2-methoxyethanol (C_3_H_8_O_2_), respectively. First, a certain amount of ZrOCl_2_·8H_2_O and GdN_3_O_9_·6H_2_O were dissolved in C_3_H_8_O_2_ and stirred with a magnetic stirrer for 3 h at room temperature to ensure complete dissolution. Next, the GdN_3_O_9_·6H_2_O solution was added dropwise to the ZrOCl_2_·8H_2_O solution at a certain ratio (molar percentage Gd/(Gd + Zr) = 15%) to form a Gd-doped ZrO_2_ precursor solution with a 15% doping concentration. The precursor concentration was maintained at 0.2 M. Then, a certain amount of hydrogen peroxide (H_2_O_2_) was added to the precursor solution to promote hydrolysis and condensation reaction, and the solution was stirred at room temperature for 8 h until it became clear and transparent. Finally, the precursor solution was aged for 72 h. To obtain clean and impurity-free precursors, the solution was filtered through a 0.22 μm PTFE syringe filter before spin-coating to facilitate the process. The process of preparing the precursor solution has been mentioned in previous studies, and thus it has very good versatility.^22–24^

### 2.2 Thin film deposition

ZrGdO_*x*_ composite films were prepared on n-type Si substrates with a resistivity of 1–10 Ω cm using the sol–gel method. For substrate cleaning, the Si substrate was ultrasonically cleaned in deionized water, acetone, and anhydrous ethanol for 15 minutes to remove organic compounds and impurities from the surface. To remove the natural oxides, the Si substrate was subsequently ultrasonically cleaned in a 1% HF solution for 3 minutes, followed by another 15 minutes ultrasonic cleaning in deionized water to remove residual cleaning reagents. Finally, the Si substrate was dried in an N_2_ environment. For thin film preparation, the ZrGdO_*x*_ precursor solution was spin-coated at 5000 rpm for 30 seconds, followed by baking at 200 °C for 5 minutes to remove the organic solvent and cure the film. These steps were repeated several times until the desired film thickness was achieved. Before the heat treatment, all ZrGdO_*x*_ films were exposed to UVO radiation for 10 minutes. Finally, the film samples were annealed in a tube furnace in an air environment at 300 °C, 400 °C, 500 °C, and 600 °C, respectively, for 1 h. The annealed samples were labeled as 300 °C, 400 °C, 500 °C, and 600 °C, respectively.

### 2.3 Thin film characterization

The thermal behavior of the ZrGdO_*x*_ thin film samples was analyzed using a Mettler thermogravimetric analysis (TGA) at a heating rate of 10 °C min^−1^ to determine the annealing temperature range. The structural information was investigated using an X-ray diffractometer (XRD) with a Cu Kα X-ray source. The XRD parameters included an accelerating voltage of 40 kV, a scanning range of 5–80°, and a step rate of 5° per min. The transmittance and optical band gap values of the samples were analyzed using an ultraviolet-visible spectrometer (UV-vis). The film thickness was estimated from the sample cross-section using a scanning electron microscope (SEM). Atomic force microscopy (AFM) was used to study the surface morphology and measure the surface roughness. The chemical composition and interfacial properties of the ZrGdO_*x*_ films were investigated by X-ray photoelectron spectroscopy (XPS), where all the XPS data were corrected for the C 1s peak at a binding energy of 284.8 eV.

### 2.4 Preparation and testing of MOS devices

**Fig. 1:**
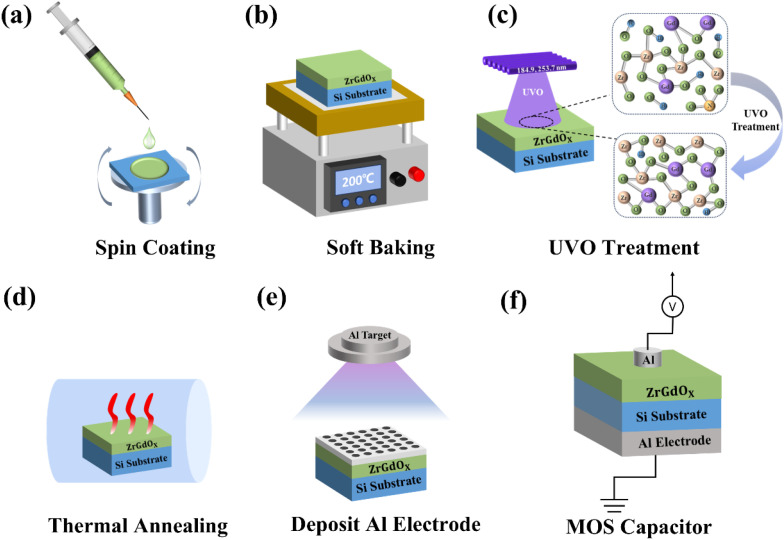
Preparation processes of the ZrGdO_*x*_ films and structured Al/ZrGdO_*x*_/Si MOS capacitors.

To investigate the electrical properties of ZrGdO_*x*_ films, MOS capacitors with the structure of Al/ZrGdO_*x*_/Si were prepared. The preparation flow of ZrGdO_*x*_ films and MOS capacitors is shown in Fig. 1. Al electrodes with an area of 1.521 × 10^−3^ cm^2^ were deposited on the ZrGdO_*x*_ films using magnetron sputtering with a shade mask in a vacuum environment. To achieve ohmic contact and reduce contact resistance, Al electrodes were also deposited on the backside of the Si substrate with a sputtering power of 50 W, a sputtering time of 600 s, and a thickness of approximately 100 nm. The capacitance–voltage (*C*–*V*) and leakage current–density–voltage (*J*–*V*) characteristics of the MOS capacitors were measured using a semiconductor analyzer in combination with a cascade probe stage at a high frequency of 1 MHz. It is important to note that short and open circuit calibrations are performed prior to the actual testing, and all electrical tests are performed at room temperature, in a dark box.

## 3. Results and discussion

### 3.1 Thermal behavior analysis

**Fig. 2:**
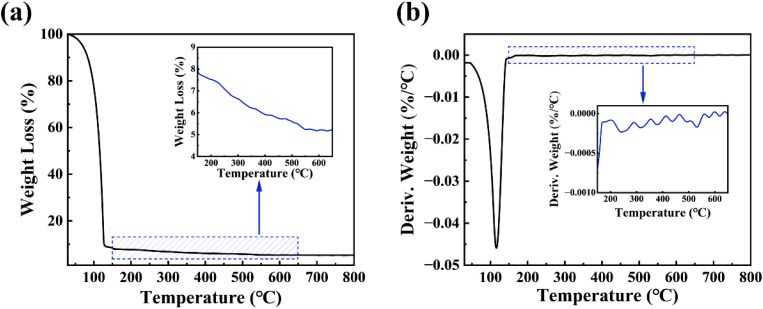
(a) TG and (b) DTG curves of ZrGdO_*x*_ precursor.

The thermogravimetric (TG) curve of the ZrGdO_*x*_ precursor is shown in Fig. 2a, which characterizes the conversion process from the precursor solution to the ZrGdO_*x*_ dry gel. The significant mass loss below 150 °C is attributed to the evaporation of the organic solvent,^25^ as the boiling point of the solvent used in this experiment is around 120 °C. This is confirmed by the sharp peak at 120 °C in the differential thermogravimetric (DTG) curve in Fig. 2b. Furthermore, the inset of Fig. 2a shows that the weight loss above 150 °C can be expressed as decomposition of the organic ligand and formation of metal–oxygen–metal frameworks, leading to a denser film and reduced impurities.^26^ There is no significant weight loss above 550 °C, indicating that the precursor solution completes its transformation at about 550 °C. In the inset of Fig. 2b, a smaller peak appears at about 540 °C, indicating the formation of the ZrGdO_*x*_ lattice. To explore the effect of annealing temperature on the Gd-doped ZrO_2_ thin films, the samples were annealed at 300, 400, 500, and 600 °C for 1 h in this experiment.

### 3.2 Microstructure analysis

**Fig. 3:**
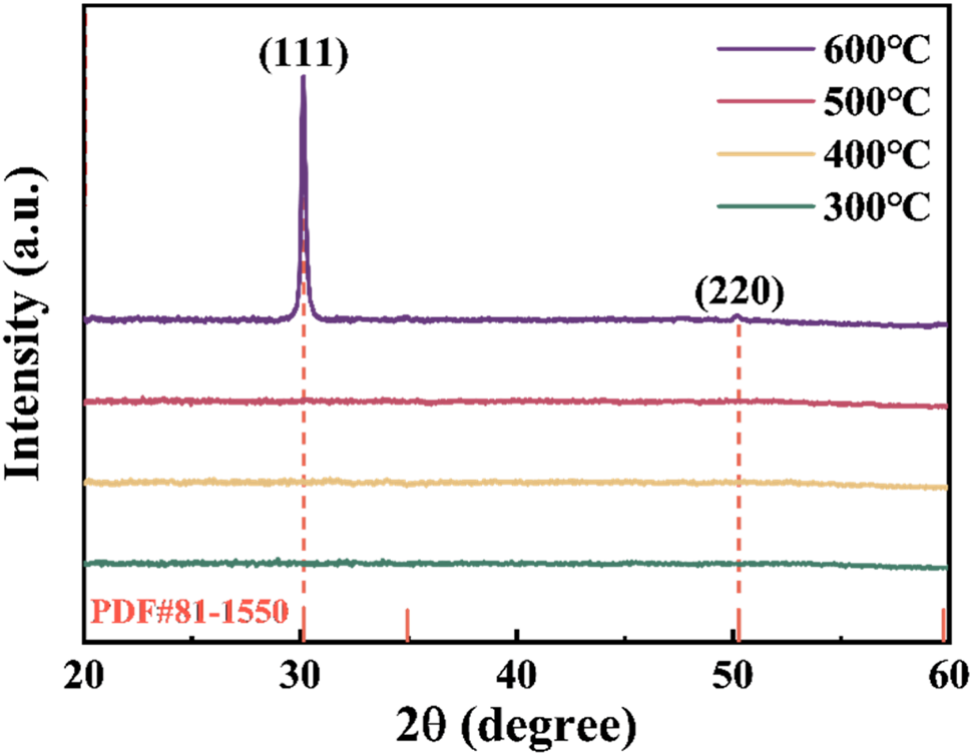
XRD pattern of ZrGdO_*x*_ thin films at different annealing temperatures.

Fig. 3 shows the XRD pattern of ZrGdO_*x*_ films at different annealing temperatures. As the annealing temperature increases from 300 °C to 500 °C, no diffraction peaks are observed, indicating that the ZrGdO_*x*_ films remain in the amorphous state. However, when the annealing temperature reaches 600 °C, diffraction peaks appear at 2*θ* = 30.2° and 50.3° for the ZrGdO_*x*_ film. According to the PDF#81-1550 card, these two diffraction peaks correspond to the (111) and (220) crystal plane, respectively. It has been reported that pure ZrO_2_ films typically crystallize at about 450 °C.^27^ These results clearly indicate that the introduction of an appropriate amount of Gd_2_O_3_ into ZrO_2_ effectively inhibits the crystallization of ZrO_2_ films, resulting in a delay of at least about 100 °C in the onset of crystallization. This is an exciting result. In future practical production processes, this advantage can be utilized to modify the microstructure of the materials through Gd doping, thereby reducing the gate leakage current of electronic devices and improving their long-term stability. Additionally, it is worth investigating the relationship between the changes in microstructure and the enhancement of dielectric properties. It is well known that for MOS electronic devices, amorphous oxide films are more suitable as gate dielectric layers than crystalline films. This is because crystalline films have a large number of grain boundaries, which act as capture and scattering centers according to the grain boundary charge capture model, leading to high gate leakage currents, reduced insulation, and increased power consumption in the device.^28^

### 3.3 Chemical bonding state analysis

**Fig. 4:**
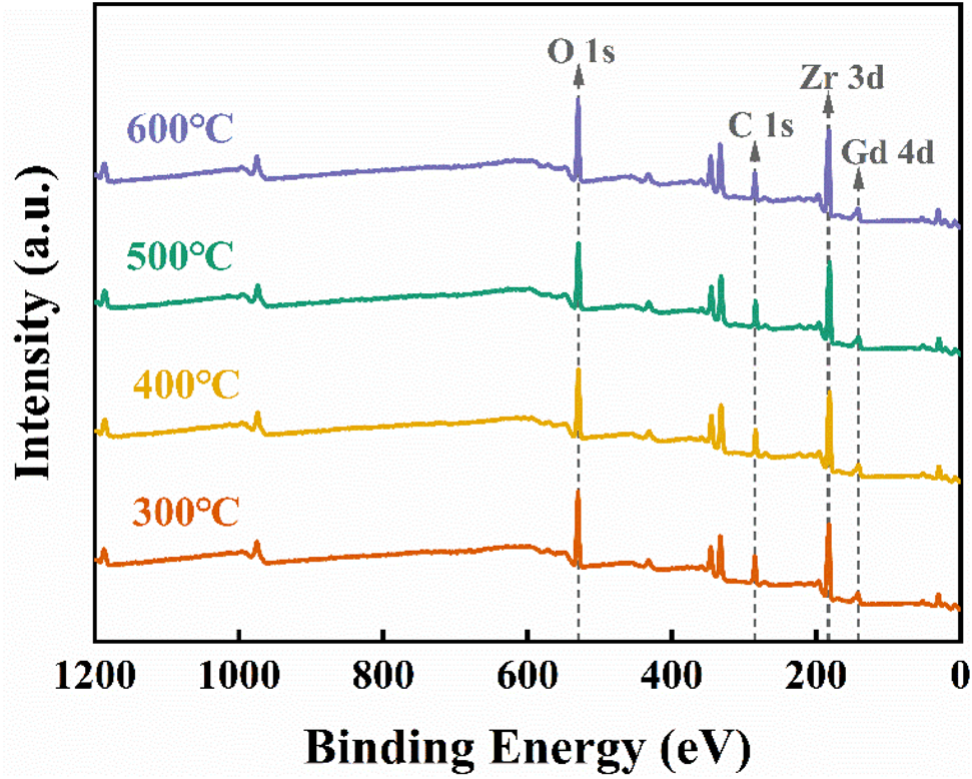
XPS full spectrum of ZrGdO_*x*_ thin films.

The chemical composition of the ZrGdO_*x*_ composite films was studied using XPS measurements, as shown in Fig. 4. To avoid experimental errors, all XPS data were charge-shifted and corrected to the binding energy of C 1s at 284.8 eV. Fig. 4 displays the wide-energy-range XPS spectra of all samples with respect to the annealing temperature. It can be noted that the signals of the elements C, O, Zr, and Gd are detected, indicating that the element Gd has been successfully doped into the ZrO_2_ gate medium. Additionally, no signals from other impurity elements were detected, confirming the absence of contamination.

**Fig. 5:**
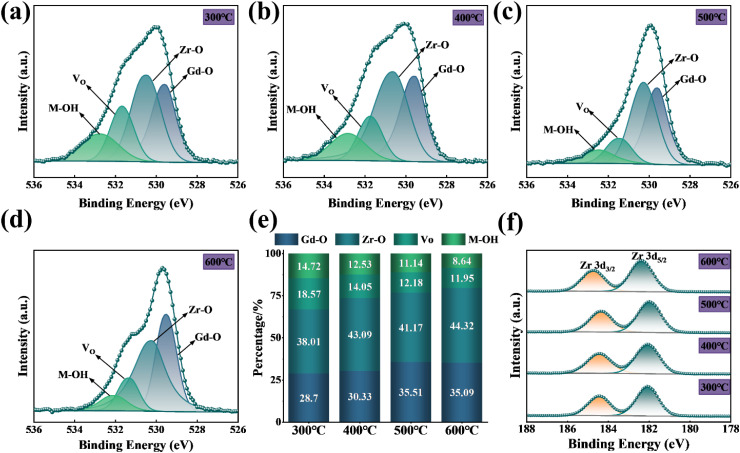
O 1s spectra of ZrGdO_*x*_ films annealed at (a) 300 °C, (b) 400 °C, (c) 500 °C, (d) 600 °C, (e) atomic percentages of the oxygen components in each sample, (f) XPS spectra of Zr 3d.

The temperature-dependent XPS spectra of O 1s in ZrGdO_*x*_ composite films are presented in Fig. 5a–d. The O 1s spectra can be fitted by Gaussian to four subpeaks, centered at 529.58, 530.26, 531.42, and 532.45 eV, respectively. The two subpeaks at 529.58 and 530.26 eV correspond to gadolinium–oxygen bond (Gd–O) and zirconium–oxygen bond (Zr–O), both of which are associated with oxygen in the metal oxide lattice.^13,29,30^ The subpeak located at 531.42 eV correspond to oxygen vacancies (*V*_O_) in the lattice,^28^ while the subpeak at 532.45 eV is attributed to hydroxyl groups and water adsorbed in air (M–OH).^22^ To better understand the changes in each oxygen component with annealing temperature in the XPS pattern of O 1s, the specific percentages of each oxygen component were extracted, as shown in Fig. 5e. As the annealing temperature increases from 300 °C to 600 °C, the proportion of metal–oxygen bonds (M–O) in the ZrGdO_*x*_ composite films increases from 66.71% to 79.41%, enhancing the capacitance and reducing the leakage current. The proportions of *V*_O_ and M–OH decrease to 11.95% and 8.64%, respectively. These results indicate that high-temperature annealing helps to eliminate oxygen vacancies and enhance dehydroxylation, promoting the conversion of metal hydroxides to metal oxides and forming metal–oxygen frameworks.^31^ As a gate dielectric layer in CMOS devices, the content of oxygen vacancies and hydroxyl oxygen should be kept as low as possible. This is because these components create trap states in the film's forbidden band, leading to increased gate leakage current and decreased breakdown voltage.^32^ Therefore, higher-temperature annealing treatment is an effective method to control the content of oxygen vacancies and hydroxyl oxygen, resulting in high-quality films.

To obtain more information about the chemical bonding states, Fig. 5f shows the evolution of the Zr 3d core energy levels as a function of the annealing temperature. The Zr 3d peaks of all thin film samples were divided into two peaks (Zr 3d_3/2_ and Zr 3d_5/2_) with a splitting energy of about 2.4 eV.^7^ For the ZrGdO_*x*_ composite films annealed at 300 °C, the centers of the Zr 3d_5/2_ and Zr 3d_3/2_ double peaks are located at 182.08 and 184.46 eV, respectively. When the annealing temperature is increased to 500 °C, the centers of the Zr 3d_5/2_ and Zr 3d_3/2_double peaks were located at 181.99 and 184.36 eV, respectively, indicating a trend towards lower binding energies. This is attributed to the fact that Zr, with a higher electronegativity than Gd, provides electrons for the Gd–O bond during annealing, generating Gd–O bonds with lower dissociation energy first.^13,33^ However, as the annealing temperature increases to 600 °C, the Zr 3d double peaks move towards higher binding energies again, with the centers of the Zr 3d_5/2_ and Zr 3d_3/2_ double peaks located at 182.36 eV and 184.74 eV, respectively. This phenomenon is attributed to the reaction of Zr and Gd atoms with O atoms at high temperatures to form the ZrGdO_*x*_ structure.^32^ Additionally, it is believed that the ZrGdO_*x*_ composite films crystallize at high temperatures, resulting in a shift of the binding energy to higher direction, which is also consistent with the previous XRD results.^34^

### 3.4 Optical characterization and energy band shift analysis

**Fig. 6:**
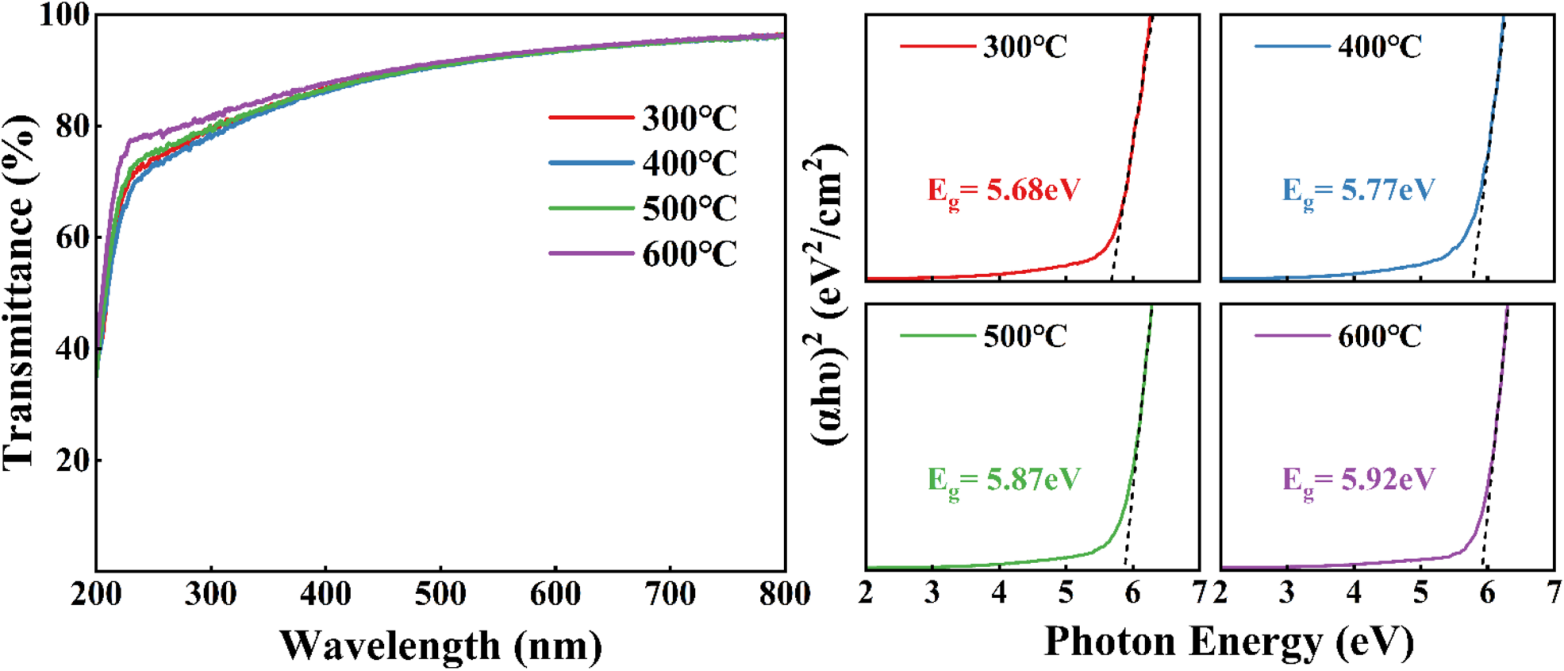
(a) Transmittance and (b) optical band gaps spectra of ZrGdO_*x*_ thin films at various annealing temperatures.

To investigate the optical properties of the ZrGdO_*x*_ composite films, the optical transmittance of all samples deposited on the quartz substrate with different annealing temperatures was measured as shown in Fig. 6a. The results indicate that all samples exhibit an average optical transmittance of over 80% in the visible region, which implies that ZrGdO_*x*_ composite films have great potential for applications in transparent electronics. The energy band shift is a critical factor in evaluating the suitability of semiconductor materials for use as high-*k* gate dielectrics in CMOS devices. Therefore, the valence band (VB) and conduction band (CB) shifts of the ZrGdO_*x*_ composite films were determined after measuring the energy band gap (*E*_g_).^35^ According to the light absorption theory, the *E*_g_ values of all samples can be determined by Tauc's formula:^23^

1 *αhν* = *A*(*hν* − *E*_g_)^*m*^

where *α* is the absorption coefficient, *hν* is the photon energy, *A* is a constant, and *E*_g_ is the optical band gap. The exponent *m* depends on the type of optical jump between the valence and conduction bands, with *m* = 1/2 for direct jump and *m* = 2 for indirect jump. Given that ZrO_2_ undergoes a direct transition, *m* should be 1/2, and therefore it is necessary to plot (*αhν*)^2^ with respect to *hν*. The *E*_g_ values can be determined by linear extrapolation when (*αhν*)^2^ = 0, corresponding to *hν* = *E*_g_. Fig. 6b illustrates the variation of *E*_g_ with different annealing temperatures. As the annealing temperature increases from 300 °C to 600 °C, the *E*_g_ values are 5.68, 5.77, 5.87, and 5.92 eV, respectively. The increase in *E*_g_ with annealing temperature can be attributed to the reduction in defects and disorder within the films, which decreases the density of localized states in the energy band structure. As a result, the decrease in localized states leads to an increase in *E*_g_.^36^Table 1 lists the *E*_g_ values of ZrO_2_ materials involved in recent studies. It can be observed that the *E*_g_ of Gd-doped ZrO_2_ in this experiment is larger than that of pure ZrO_2_ in previous studies, which is attributed to the d–d coupling of electrons in the Zr 5d and Gd 5d antibonding states.^40^ Furthermore, among the various doping elements, a moderate amount of Gd exhibits the most significant ability to increase the *E*_g_ value of ZrO_2_.

**Table 1:** Band gap values of relevant ZrO_2_ materials in recent years

Sample	Experimental variable	*E*_g_ (eV) of pure ZrO_2_	*E*_g_ (eV) of the best sample	Year	Ref.
B-doped ZrO_2_	B-doping concentration	5.5	5.17	2015	37
ZrO_2_	Annealing temperature	5.3	5.7	2016	38
Gd-doped ZrO_2_	Gd-doping concentration	5.65	5.83	2016	13
Y-doped ZrO_2_	Y-doping concentration	4.91	4.92	2017	39
ZrO_2_	Annealing temperature	5.8	—	2018	18
Al-doped ZrO_2_	Annealing temperature	5.77	5.79	2022	7
La-doped ZrO_2_	La-doping concentration	5.68	5.71	2023	22
Gd-doped ZrO_2_	Annealing temperature	—	5.87	2024	This work

The valence band offset (Δ*E*_v_) of the ZrGdO_*x*_ composite film was determined by measuring the difference in the valence band maximum (VBM) between the dielectric layer and the substrate as follows:^41^

**Fig. 7:**
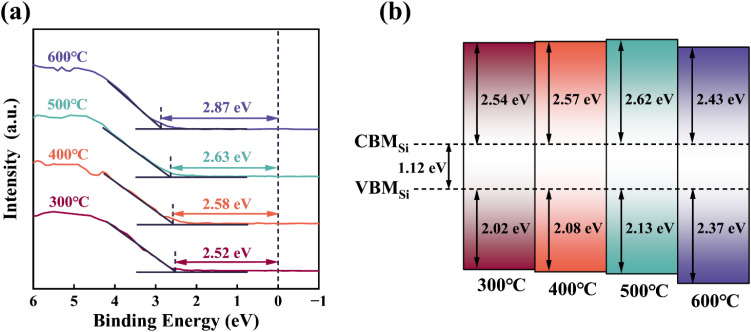
(a) Valence band spectra of ZrGdO_*x*_ thin films, (b) schematic energy bands of ZrGdO_*x*_/Si gate stacks.

2 Δ*E*_v_(ZrGdO_*x*_/Si) = *E*_v_(ZrGdO_*x*_) − *E*_v_(Si)

where *E*_v_(ZrGdO_*x*_) is the maximum valence band value of the thin film sample and *E*_v_(Si) is the maximum valence band value of the Si substrate of 0.5 eV. Fig. 7a shows the VBM of ZrGdO_*x*_ composite films at different annealing temperatures. Using the above eqn (2), the Δ*E*_v_ values for the samples annealed at 300, 400, 500, and 600 °C can be obtained as 2.02, 2.08, 2.13, and 2.37 eV, respectively.

The conduction band offset (Δ*E*_c_) for all samples can be determined by the difference between the band gap values of the films and the Δ*E*_v_, the band gap value of Si substrate, using the following eqn (3):

3 Δ*E*_c_(ZrGdO_*x*_/Si) = *E*_g_(ZrGdO_*x*_) − Δ*E*_v_(ZrGdO_*x*_/Si) − *E*_g_(Si)

where *E*_g_(ZrGdO_*x*_) and *E*_g_(Si) are the bandgap values of the thin films and the Si substrate, respectively. The Δ*E*_c_ values are calculated to be 2.54, 2.57, 2.62, and 2.43 eV for the samples annealed at 300, 400, 500, and 600 °C, respectively. Based on the above calculations, Fig. 7b schematically illustrates the energy bands and energy band offsets for the ZrGdO_*x*_/Si gate-stacked structures at different annealing temperatures. It can be noticed that all Δ*E*_v_ and Δ*E*_c_ values exceed 1 eV, with the sample annealed at 500 °C showing the largest Δ*E*_c_ value. A sufficiently large barrier height is crucial for blocking electrons and holes, inhibiting carrier transport, and reducing gate leakage current, making the ZrGdO_*x*_ composite films annealed at 500 °C suitable for practical applications.

### 3.5 Micro-morphological analysis

**Fig. 8:**
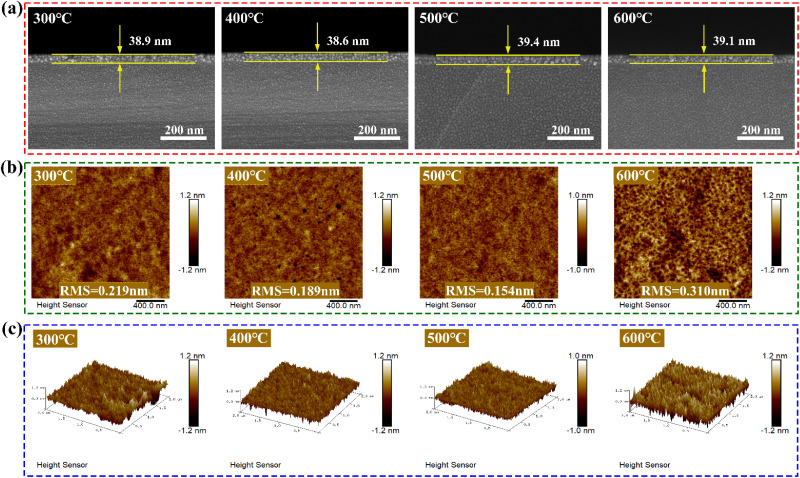
(a) Cross-sectional SEM patterns of ZrGdO_*x*_ thin films, (b) 2D and (c) 3D AFM maps of the samples at different annealing temperatures, respectively.

Fig. 8a displays the SEM cross sections of the ZrGdO_*x*_ composite thin films after different annealing heat treatments. The SEM images reveal a distinct demarcation between the thin film and the Si substrate. After measuring the actual film thicknesses, the ZrGdO_*x*_ composite films annealed at 300, 400, 500, and 600 °C had thicknesses of approximately 38.9, 38.6, 39.4, and 39.1 nm, respectively. These measurements are crucial for calculating the electrical parameters of MOS capacitors. For CMOS devices, performance is highly dependent on the surface quality of the gate dielectric film.

Fig. 8b and c present the 2D and 3D AFM patterns of the ZrGdO_*x*_ composite films at different annealing temperatures, with a scanning range of 2 × 2 μm. The root-mean-square (RMS) roughness values of the films annealed at 300, 400, 500, and 600 °C were 0.219, 0.189, 0.154, and 0.310 nm, respectively. The average RMS value of the ZrGdO_*x*_ composite films prepared in the current work was only 0.218 nm, indicating that the films were uniformly smooth. This is inextricably linked to the UV ozone-assisted treatment process, as it has been shown that a suitable UV ozone treatment time can effectively clean the oxide surface.^42^ As the annealing temperature increased from 300 °C to 500 °C, the RMS values continued to decrease, likely due to the removal of residual organic groups and the formation of a dense metal oxide framework. However, the RMS value of the sample annealed at 600 °C was higher than the others, which was attributed to the fact that this temperature induced crystallinity and degraded film quality, as confirmed by XRD results.^43^ Maintaining a smooth and uniform surface in ZrGdO_*x*_ composite films as gate dielectrics is essential to minimize interfacial trap density and carrier scattering centers. In this paper, the surface smoothness of the films prepared using the UV ozone-assisted solution method is comparable to that of films produced by magnetron sputtering and atomic layer deposition.^44,45^

### 3.6 Electrical characterization

**Fig. 9:**
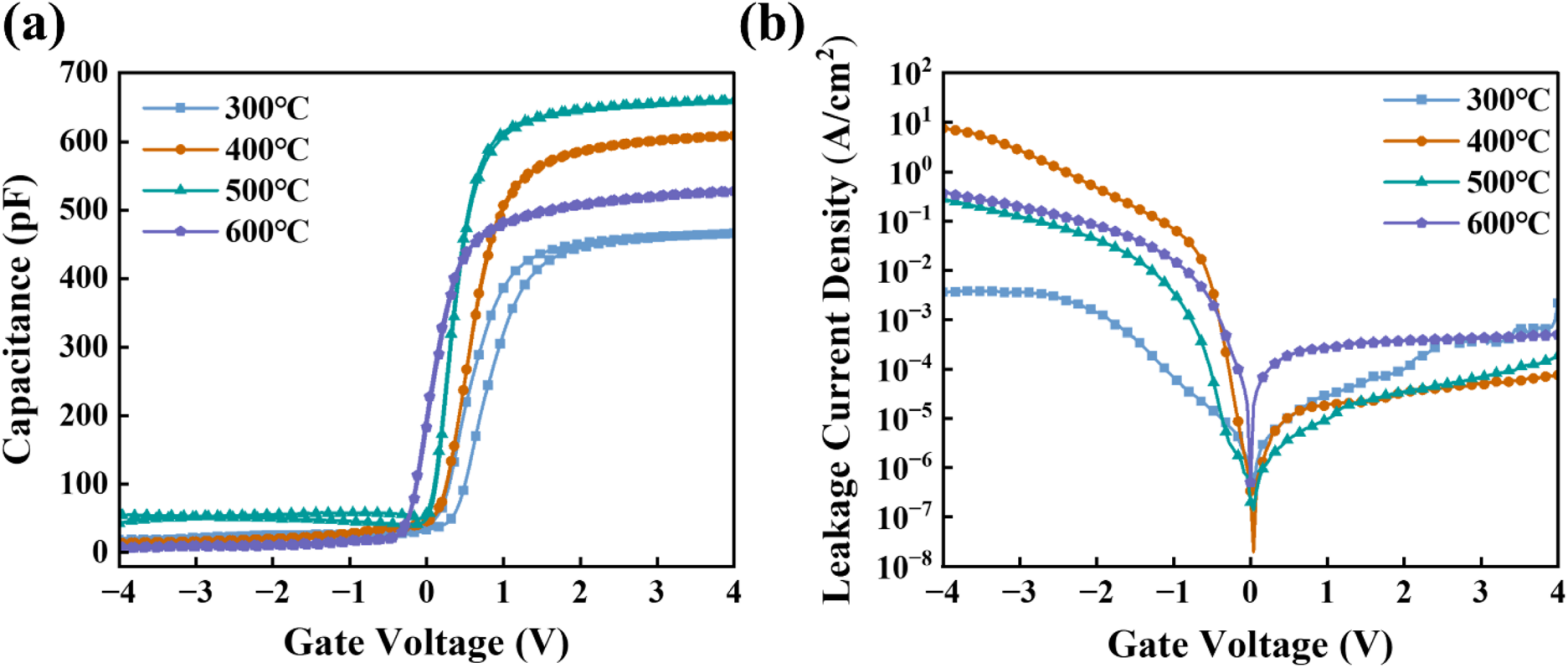
(a) *C*–*V* and (b) *J*–*V* curves of the MOS capacitors based on ZrGdO_*x*_ films.

To evaluate the electrical characteristics of the ZrGdO_*x*_ composite films as gate dielectrics, MOS capacitors with the structure of Al/ZrGdO_*x*_/Si were fabricated. Fig. 9a presents typical *C*–*V* characteristic curves at high frequency (1 MHz) for MOS capacitors based on ZrGdO_*x*_ composite films annealed at different temperatures. All samples were scanned from a gate voltage of −4 V to +4 V and then inversely, to determine their hysteresis. According to Fig. 9a, the ZrGdO_*x*_ composite films annealed at 300, 400, 500, and 600 °C exhibited cumulative capacitances (C_ox_) of 466, 609, 660, and 528 pF, respectively, at a gate voltage of +4 V. The dielectric constant (*k*) and the equivalent oxide thickness (EOT) of the ZrGdO_*x*_ composite films can be calculated using the following eqn (4) and (5):4
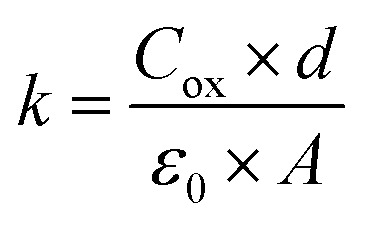
5
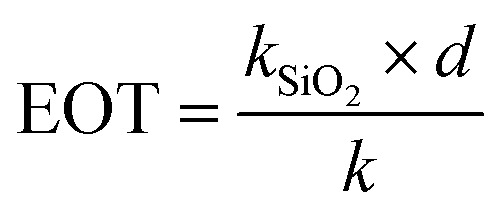
where *C*_ox_ is the cumulative capacitance of the sample, *d* is the actual physical thickness of the film, *ε*_0_ is the vacuum dielectric constant, *A* is the area of the Al electrode, and *k*_SiO_2__ is the dielectric constant of SiO_2_. The *k* values of the ZrGdO_*x*_ composite films annealed at 300, 400, 500, and 600 °C were calculated to be 13.5, 17.5, 19.3, and 15.3, respectively. Additionally, the EOT values of these films were 11.2, 8.6, 8.0, and 9.9 nm, respectively. For the sample annealed at 500 °C, the smallest EOT (8.0 nm) and the largest *k* value (19.3) were obtained. Notably, as the annealing temperature increased to 500 °C, the EOT decreased continuously, and the *k* value increased. However, when the annealing temperature was raised to 600 °C, the EOT suddenly increased and the *k* value decreased. Two possible reasons for this phenomenon are: first, the reaction between the ZrGdO_*x*_ composite film and the Si substrate during high-temperature annealing, which generates a low-*k* interfacial layer, increasing the EOT and decreasing the *k* value. The second reason could be the degradation of the interfacial quality at higher annealing temperatures, confirmed by AFM data.

Furthermore, the flat-band capacitance (*C*_fb_) of the ZrGdO_*x*_ composite films annealed at 300, 400, 500, and 600 °C can be calculated, and the flat-band voltage (*V*_fb_), and hysteresis (Δ*V*_fb_) can be determined sequentially from the *C*–*V* curves. In addition, the equivalent oxide charge density (*Q*_ox_), and the boundary trap oxide charge density (*N*_bt_) can be calculated by the following eqn (6)–(8):^46^6
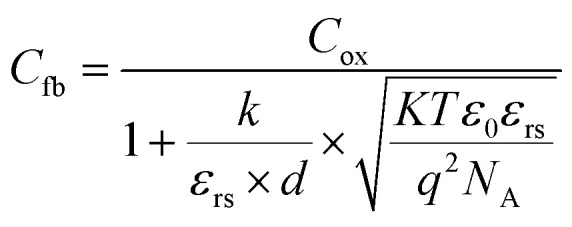
7
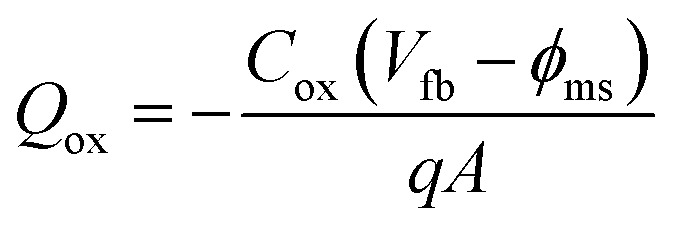
8
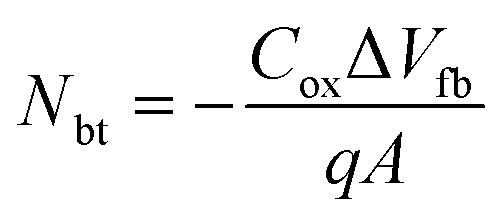
where *k* is the dielectric constant of the ZrGdO_*x*_ composite film, *ε*_rs_ is the dielectric constant of the Si substrate, *d* is the actual physical thickness of the ZrGdO_*x*_ composite film, *K* is Boltzmann's constant, *T* = 300 K, *ε*_0_ is the vacuum dielectric constant, *N*_A_ is the carrier concentration, *φ*_ms_ is the difference between the work function of the Al electrode and the Si substrate, *A* is the area of the Al electrode, and *q* is the charge of the electron.

The detailed electrical parameters are shown in Table 2. The *V*_fb_ is always positive, indicating the presence of negative trap charge in the film, which may originate from OH^−^ in the residual organic solvent in the dielectric.^23^ Additionally, the absolute value of *V*_fb_ decreases with increasing annealing temperature, and the *Q*_ox_ obtained from *V*_fb_ decreases from 1.76 × 10^12^ cm^−2^ to 5.64 × 10^11^ cm^−2^, suggesting that the films at higher annealing temperature contain fewer defects and traps. From Table 2, it can be clearly seen that the ZrGdO_*x*_ composite film annealed at 500 °C exhibits the smallest Δ*V*_fb_ (0.01 V), indicating a minimum of oxide trapping charge in the films. Since *N*_bt_ depends on Δ*V*_fb_ in a certain degree, the sample annealed at 500 °C has the smallest *N*_bt_ (6.5 × 10^10^ cm^−2^) by rigorous calculation.

**Table 2:** Specific electrical parameters of the MOS capacitors

Sample	*C*_ox_ (pf)	EOT (nm)	*k*	*C*_fb_ (pf)	*V*_fb_ (V)	Δ*V*_fb_ (V)	*Q*_ox_ (cm^−2^)	*N*_bt_ (cm^−2^)
300 °C	466	11.2	13.5	211.5	0.72	0.22	1.76 × 10^12^	4.2 × 10^11^
400 °C	609	8.6	17.5	236.7	0.49	0.02	1.73 × 10^12^	5.0 × 10^10^
500 °C	660	8.0	19.3	244.5	0.26	0.01	1.25 × 10^12^	2.7 × 10^10^
600 °C	528	9.9	15.3	224.0	0.06	0.03	5.64 × 10^11^	6.5 × 10^10^

Fig. 9b illustrates the *J*–*V* characteristic curves of the MOS capacitor with structure Al/ZrGdO_*x*_/Si. It is evident that the *J*–*V* curves are asymmetric about 0 V, attributed to the different carrier supply under different gate voltage injection modes. Different energy bands in these injection modes result in a much smaller leakage current density under substrate injection compared to gate injection for the same absolute gate voltage value.^47^ The composite film annealed at 500 °C exhibited the lowest leakage current density (9.61 × 10^−6^ A cm^2^) at a gate voltage of 1 V. However, when the annealing temperature was increased to 600 °C, the leakage current density of the sample drastically increased to, attributed to microstructural alteration, interfacial quality deterioration, and defect increases. In conclusion, the composite film annealed at 500 °C demonstrated the best electrical properties.

Table 3 lists the best dielectric properties of some high-*k* materials in recent years, comparing them with the dielectric parameters of the composite films annealed at 500 °C in this work. It is found that compared to expensive and complex process methods, the gate dielectric films prepared by UVO-assisted sol–gel method, which is a simple and efficient approach, exhibit better electrical properties than other gate dielectrics. And this is a promising result. Therefore, the excellent dielectric properties of the gate dielectric films, combined with the facile and low-cost preparation process, offer great potential for the future development of low-cost and high-performance MOS devices.

**Table 3:** Electrical parameters of different high-*k* dielectric materials reported in recent years

High-*k* materials	Preparation method	Annealing temperature	Dielectric constant (*k*)	Leakage current (A cm^−2^)	Year	Ref.
B-doped	Sol–gel	150 °C	10.5	9.1 × 10^−5^	2015	37
Ti-doped	Sputtering	—	39.92	1.39 × 10^−5^	2016	48
Gd-doped	Sol–gel	400 °C	11.4	— × 10^−6^	2017	49
Y-doped	Sputtering	600 °C	11.28	9.69 × 10^−4^	2018	16
	Sputtering	600 °C	21.2	4.94 × 10^−3^	2019	32
Al-doped	ALD	200 °C	8.1	6.2 × 10^−7^	2020	50
	Sputtering	430 °C	—	0.79 × 10^−4^	2021	51
La-doped	Sol–gel	500 °C	11.5	6.3 × 10^−6^	2023	22
Gd-doped	Sol–gel	500 °C	19.3	9.61 × 10^−6^	2024	This work

### 3.7 Leakage current mechanism analysis

**Fig. 10:**
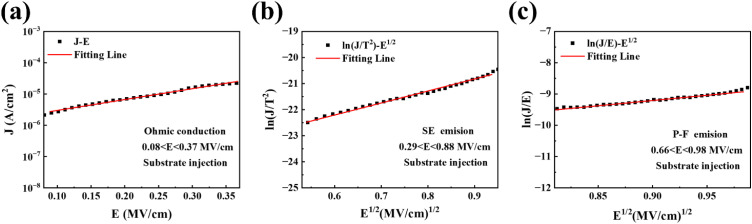
(a) Ohmic conduction, (b) SE emission, and (c) P–F emission plots of Al/ZrGdO_*x*_/Si capacitors in substrate injection mode.

Since gate leakage current increases the power consumption of electronic devices, the leakage current mechanism was analyzed for composite films annealed at 500 °C to better understand the generation of leakage current. Due to the complexity of leakage current conduction in thin films, this paper mainly analyzes the leakage current using three conduction mechanisms: ohmic conduction, Schottky emission (SE), and Pool–Frenkel (P–F). In Fig. 10a, a good linear relationship between *J* and *E* is observed in the field strength range of 0.08 < *E* < 0.37 MV cm^−1^, indicating that ohmic conduction is the dominant conduction mechanism in this range. Ohmic conduction occurs due to the movement of free particles in the conduction or valence bands of the dielectric at low voltages. Fig. 10b shows a good linear relationship between and *E*^1/2^ within a field strength range of, indicating that Schottky emission is the dominant conduction mechanism in this range. Schottky emission results from thermal excitation, which provides electrons with energy sufficient to overcome the potential barrier between the Si substrate and the dielectric, allowing them to enter the gate dielectric layer. From Fig. 10c, it is observed that in the field strength range of in the middle to high electric field strength, the P–F emission satisfies the linear relationship between *E*^1/2^ and, indicating that P–F conduction is dominant at this time. In conclusion, the leakage current conduction mechanism of ZrGdO_*x*_ composite films annealed at 500 °C transitions from ohmic conduction to Schottky emission to Pool–Frenkel emission as the electric field strength increases.

## 4. Conclusion

ZrGdO_*x*_ composite films were successfully prepared on n-type Si substrates using UVO-assisted sol–gel method. The annealing temperature range of the ZrGdO_*x*_ composite films was first determined through TG analysis. Subsequently, the effects of different annealing temperatures on the microstructure, chemical bonding state, optical properties, surface morphology, and electrical properties of the ZrGdO_*x*_ composite films were investigated by using XRD, XPS, UV-vis, SEM, AFM, and semiconductor analyzers. The XRD results showed that doping with Gd element increased the crystallization temperature of the ZrO_2_ films. Additionally, the ZrGdO_*x*_ composite films heat-treated at 500 °C remained in an amorphous state, which is ideal for use as a gate insulating layer in CMOS device. XPS analysis revealed that at 500 °C, the ZrGdO_*x*_ composite films had a relatively high proportion of M–O bonds (76.68%) and low proportions of *V*_O_ (12.18%) and M–OH (11.14%), indicating that this annealing temperature can promote the formation of a dense metal–oxygen framework. The optical transmittance and band gap values of the ZrGdO_*x*_ composite films at various heat treatment temperatures were obtained *via* UV-vis spectroscopy, and the energy band shifts were determined combined from the valence band spectra. The sample annealed at 500 °C exhibited a large band gap value (*E*_g_ = 5.87 eV) and conduction band offset (Δ*E*_c_ = 2.62 eV), which helps to suppress carrier transport and leakage current generation. The AFM results indicated that the thin films, treated with UV ozone, had smooth surfaces with RMS values as low as 0.154 nm. The MOS capacitor with the structure Al/ZrGdO_*x*_/Si was tested and analyzed, revealing that the gate dielectric film exhibited the best electrical properties when the annealing temperature was 500 °C. At this temperature, the film had the highest dielectric constant (*k* = 19.3), the smallest boundary trap oxide charge density (*N*_bt_ = 2.7 × 10^10^ cm^−2^), the smallest hysteresis (Δ*V*_fb_ = 0.01 V), and the lowest gate leakage current density (*J* = 9.61 × 10^−6^ A cm^−2^). Finally, the leakage current mechanism was analyzed for a MOS capacitor with a 500 °C annealed ZrGdO_*x*_ composite film as the gate dielectric layer. The leakage current conduction mechanisms were found to be Ohmic conduction, Schottky emission, and Pool–Frenkel conduction at low, medium, and medium–high electric field strengths, respectively.

## Data availability

The data supporting the findings of this study are available from the corresponding author upon reasonable request.

## Author contributions

Chaozhong Guo: data curation (lead); formal analysis (lead); investigation (lead); methodology (lead); writing – original draft (lead); writing – review & editing (lead). Kamale Tuokedaerhan: supervision (lead); resources (lead); conceptualization (lead); funding acquisition (lead). Xiangqian Shen: methodology (supporting). Yerulan Sagidolda: resources (supporting). Zhambyl Azamat: resources (supporting).

## Conflicts of interest

There are no conflicts to declare.
